# Thriving under Stress: Selective Translation of HIV-1 Structural Protein mRNA during Vpr-Mediated Impairment of eIF4E Translation Activity

**DOI:** 10.1371/journal.ppat.1002612

**Published:** 2012-03-22

**Authors:** Amit Sharma, Alper Yilmaz, Kim Marsh, Alan Cochrane, Kathleen Boris-Lawrie

**Affiliations:** 1 Department of Veterinary Biosciences, Ohio State University, Columbus, Ohio, United States of America; 2 Center for Retrovirus Research, Ohio State University, Columbus, Ohio, United States of America; 3 Center for RNA Biology, Ohio State University, Columbus, Ohio, United States of America; 4 Comprehensive Cancer Center, Ohio State University, Columbus, Ohio, United States of America; 5 Department of Molecular Genetics, University of Toronto, Toronto, Canada; University of Pennsylvania School of Medicine, United States of America

## Abstract

Translation is a regulated process and is pivotal to proper cell growth and homeostasis. All retroviruses rely on the host translational machinery for viral protein synthesis and thus may be susceptible to its perturbation in response to stress, co-infection, and/or cell cycle arrest. HIV-1 infection arrests the cell cycle in the G2/M phase, potentially disrupting the regulation of host cell translation. In this study, we present evidence that HIV-1 infection downregulates translation in lymphocytes, attributable to the cell cycle arrest induced by the HIV-1 accessory protein Vpr. The molecular basis of the translation suppression is reduced accumulation of the active form of the translation initiation factor 4E (eIF4E). However, synthesis of viral structural proteins is sustained despite the general suppression of protein production. HIV-1 mRNA translation is sustained due to the distinct composition of the HIV-1 ribonucleoprotein complexes. RNA-coimmunoprecipitation assays determined that the HIV-1 unspliced and singly spliced transcripts are predominantly associated with nuclear cap binding protein 80 (CBP80) in contrast to completely-spliced viral and cellular mRNAs that are associated with eIF4E. The active translation of the nuclear cap binding complex (CBC)-bound viral mRNAs is demonstrated by ribosomal RNA profile analyses. Thus, our findings have uncovered that the maintenance of CBC association is a novel mechanism used by HIV-1 to bypass downregulation of eIF4E activity and sustain viral protein synthesis. We speculate that a subset of CBP80-bound cellular mRNAs contribute to recovery from significant cellular stress, including human retrovirus infection.

## Author Summary

Retroviruses are intracellular parasites that utilize the host translation machinery to catalyze viral protein synthesis. The activity of the translation machinery fluctuates during cell cycle progression and is reduced in the G2/M phase. HIV-1 infection causes the cells to arrest in the G2/M phase, which has the potential to alter the activity of the translation machinery. Herein several lines of evidence demonstrated that lymphocyte mRNA translation is suppressed by the action of HIV-1 accessory protein Vpr. The molecular basis of translation suppression is reduced activity of the rate-limiting translation intitation factor, eIF4E. However, synthesis of the viral structural proteins is sustained and is due to the difference in composition of the viral and cellular mRNA-ribonucleoprotein complexes. Both cellular and completely spliced viral mRNAs are predominantly associated with the cytoplasmic cap binding protein, eIF4E. In contrast, unspliced HIV-1 mRNAs are predominantly associated with the components of the nuclear cap binding complex (CBC). The retention of CBC on the viral mRNAs provides a mechanism to sustain viral protein synthesis. This newly characterized interface of the virus-host-protein synthesis machinery is likely a cellular adaptation used to enable synthesis of proteins that reengage the cell cycle and facilitate recovery from stress.

## Introduction

Regulation of translation is a fundamental property of cell physiology. In dividing cells, mRNA translation is robust in the G1 growth phase, diminishes during the S phase, and is downregulated during the G2/M phase [1], [2], [3]. In the virus-infected cell, pathogen-associated molecular patterns trigger signaling cascades that downregulate mRNA translation to generate a protective, antiviral state [4], [5], [6]. This involves post-translational modification of components of the mRNA-ribonucleoprotein complexes (mRNPs) that are necessary for translation [7], [8], [9]. The resumption of protein synthesis involves loading of ribosomes at the 5′ untranslated region (UTR) of the mRNA. Determination of the mRNP components that modulate resumption of protein synthesis remains an active area of study.

The mRNP is initially formed upon addition of 7-methylguanosine to the 5′ end of the RNA, triggering binding of the nuclear cap binding complex (CBC, composed of CBP80 and CBP20) [10]. The CBC remains a component of the RNP during intron removal, deposition of the exon junction complex (EJC) and nuclear export. In the cytoplasm, the CBC is sufficient for an initial round of mRNA translation. If the ribosome encounters a termination codon before an EJC, a rearrangement of the RNP is triggered that culminates in the degradation of mRNA by nonsense mediated decay (NMD) [11]. Alternatively, if the ribosome encounters a termination codon after an EJC, a rearrangement of the RNP is triggered that circumvents NMD and the mRNA undergoes steady state translation. Part of the RNP rearrangement involves the replacement of the CBC by the cytoplasmic cap-binding protein, eIF4E, and the replacement of the nuclear polyadenylate binding protein (PABP-N) at the 3′ end of the mRNA by the cytoplasmic PABP-1. eIF4E is the rate-limiting component of the translation initiation machinery and downregulation of eIF4E activity attenuates steady state mRNA translation [1], [3], [12]. eIF4E function is regulated by posttranslational modifications. Hypophosphorylation of eIF4E and/or the eIF4E-binding proteins (4E-BPs) downregulates the activity of eIF4E by blocking interaction with eIF4G [13], [14]. In contrast, hyperphosphorylation of the 4E-BPs increases the interaction of eIF4E and eIF4G, facilitating translation initiation.

Downregulation of the eIF4E activity is a strategic tool for cells to rapidly respond to stress or alterations in cell cycle progression [1], [15], [16]. The modulation of the eIF4E activity has proven beneficial for the members of the *Picornaviridae* (e.g. poliovirus, encephalomyocarditis virus) [17]. By downregulating the activity of eIF4E, these viruses inhibit the synthesis of the host proteins while favouring synthesis of viral proteins [18]. The translation of picornaviral mRNAs persists by use of eIF4E-independent internal ribosome entry sites (IRES) [17]. Conceivably, the CBC-bound mRNAs would be capable of sustaining translation during downregulation of eIF4E-dependent translation [19].

In contrast to most host mRNAs, expression of the HIV-1 mRNA encoding the viral structural proteins is dependent upon an alternate pathway for the export of the corresponding mRNAs. While bulk mRNA export uses NXF1 as the export mediator, transport of HIV-1 unspliced and singly spliced RNAs to the cytoplasm involves use of the rRNA and tRNA export factor, CRM1 [20], [21]. At present, it is unclear whether the use of an alternate export pathway is necessary to bypass control mechanisms that block export of incompletely spliced mRNAs to the cytoplasm, or is necessary to confer a unique pattern of cytoplasmic regulation.

In addition to the interaction of the HIV-1 RNA with the host gene expression machinery, the HIV-1 protein products interact with cellular pathways to induce alterations in cell function [22]. A case in point is HIV-1 viral protein R (Vpr) which alters cell cycle progression by arresting cells in the G2/M phase [23]. Vpr-induced G2/M arrest also appears to contribute to the pathogenesis of HIV-1 based on the positive selection for Vpr in HIV-1 infected individuals and enhancement of virion production in cells [24], [25]. In light of the known interaction between the cell cycle and translation control, it is possible that mRNA translation is attenuated during viral infection [1], [3], [26]. How viral protein synthesis could be maintained under conditions of reduced host translation remains an open question. In this report, we demonstrate that the virus-induced G2/M arrest is necessary and sufficient to downregulate host protein synthesis. Furthermore, Vpr is sufficient for the observed reduction in global translation. The mechanism is the downregulation of eIF4E activity. Despite the reduced level of global translation during HIV-1 infection, synthesis of viral protein is maintained. Analysis of the components of the viral RNP revealed that, in contrast to the host cell mRNAs, HIV-1 incompletely spliced RNAs retain interaction with the CBC during translation and packaging into virions. The unique composition of the viral mRNPs may explain the sustained translation of the unspliced viral mRNA during downregulation of the host translation machinery.

## Materials and Methods

### Expression plasmids and proviruses

HIV-1^NL4-3^ was obtained from AIDS Reagent Reference Program. pHRvprIRESgfp, pHRvpr(R80A)IRESgfp, pHRvpr(Q65R)IRESgfp, pHRvpr(R80A/Q65R)IRESgfp and pNL4-3-VprX were obtained from Vicente Planelles (University of Utah) [27], [28]. pHRvprIRESgfp was digested with SalI-XhoI and the backbone was religated to generate pHR-IRESgfp. pNL4-3-VprX and pHIV-1^NL4-3^ΔVif were digested with NheI-PflMI and religated to generate ΔVifVprX. HIV-1^HXB2R-RI−^ was obtained from Eric Cohen (Universite de Montreal) [29]. The RNA probe spanning the major splice donor of HIV-1 was constructed by PCR on the template HxBRU with the primers gagSDF (5′-CGCGGATCCGAAGTAGTGTGTGCCCGTCT-3′) and gagSDR (5′-CCCAAGCTTCCCTGCTTGCCCATACTATA-3). All plasmid preparations were confirmed by DNA sequence analysis.

### Cells, infections and transfections

CEMx174 lymphocytes were grown in RPMI with 10% fetal bovine serum and 1% antibiotic/antimycotic. HEK293 cells were grown in DMEM with 10% fetal bovine serum and 1% antibiotic/antimycotic. HEK293 cells were transfected with the indicated HIV-1^NL4-3^ molecular clone and cell-free supernatants were collected and used to infect CEMx174 lymphocytes. Typically one million CEMx174 cells were incubated with 8×10^6^ pg of cell-free Gag for 2 h to generate HIV-1 producer cells. Coculture infections combined producer and naive CEMx174 cells at 1∶10 ratio and intracellular Gag production was evaluated by flow cytometry beginning at 8 h for indicated intervals. At 32 and 46 h post-infection, the cocultures were treated with nocodazole (Noc, 0.4 µg/ml) for 16 h and cyclohexamide (CHX, 100 µg/ml) for 2 h, respectively. HEK293 cells were treated with 1 µM nocadozole for 24 hours. HEK293 cells (2×10^5^) were transfected for 48 h in 6-well plates with 1 µg of indicated plasmid, FuGene 6 (Roche) following manufacturer's protocol.

### Ribosome profile analyses

Ribosomal profiles were prepared as described previously [30]. Briefly, 1×10^7^ cells were treated with 200 µM cycloheximide for 15 min at 37°C, washed in cold PBS containing 200 µM cycloheximide, collected by centrifugation at 1540 rpm for 4 min, and lysed in 450 µl gradient buffer (10 mM Hepes, 7 mM MgCl, 10 mM NaCl, 3 mM CaCl, 1 mM DTT, 0.5% NP40, supplemented with 2 µl/mL RNAsin and 1 µl/ml cycloheximide). For EDTA treatment, 30 mM EDTA was added to the PBS and gradient buffer. Lysates were layered on the linear sucrose gradients (15–47.5%) and centrifuged at 36,000 rpm for 2.25 h at 4°C in SW41 rotor (Beckman). Gradients were fractionated and A_254_ absorbance was measured using an ISCO system. Alternative fractions were collected for RNA or protein isolation. For RNA isolation, fractions were treated with an equivalent volume of 100% ethanol and tRNA (1 µg). For protein isolation, fractions were equilibrated in 10% TCA. Samples were incubated at −80°C overnight and centrifuged at 16,000 *g* for 20 min, and extracted with Trizol (Invitrogen) by the manufacturer's protocol.

To fractionate cytosolic RNPs and membrane-associated RNPs, CEMx174 cells (6×10^7^) were collected and resuspended in 110 mM KOAc, 25 mM K-HEPES (pH 7.5), 2.5 mM Mg(OAc)2, 1 mM EGTA, 1 mM DTT, 1 mM PMSF, 200 µM CHX, 50 U/mL RNasin (Promega), 150 µg/mL digitonin (Sigma) [31], incubated on ice for 5 min and centrifuged at 10,000 *g* for 10 min at 4°C. The supernatant containing soluble cytosolic RNPs was collected. The pellet containing membrane-associated RNPs was rinsed twice with ice-cold cytosol buffer lacking detergent and extracted in 400 mM KOAc, 25 mM K-HEPES (pH 7.5), 15 mM Mg(OAc)_2_, 1 mM DTT, 1 mM PMSF, 200 µM cycloheximide, 50 U/mL RNasin (Promega), 20 mg/mL digitonin. The suspension was incubated on ice for 30 min, centrifuged at 10,000 *g* for 10 min at 4°C, and the supernatant was collected. Aliquots of the preparations were reserved for immunoblotting.

### Flow cytometry

Intracellular staining of Gag with FITC conjugated anti-Gag p24 antibody (KC57-FITC, Beckman Coulter) was used to monitor the progress of viral infections. Cells were fixed and permeabilized with Cytofix/Cytoperm kit (BD Biosciences) according to manufacturer's protocol. Cell cycle analyses were performed using propidium iodide (PI) staining. Briefly, 5×10^5^ cells were incubated in hypotonic PI staining buffer (0.1% Sodium Citrate, 0.3% Triton-X, 100 µg/ml PI and 20 µg/ml RNase A) for 30 min on ice followed by flow analysis. ModFit generated the cell cycle profiles. GFP expression was analyzed on a FACS Aria cell sorter.

### Metabolic labeling

Cell cultures were rinsed and incubated for 30 min in RPMI supplemented with 10% dialyzed FBS that lacked methionine and cysteine, followed by addition of [^35^S]-cysteine/methionine (MP Biomedicals) (50 µCi/ml). After incubation for 1 hour, the cells were rinsed with PBS and lysed in 50 mM Tris pH 8.0, 0.1% SDS, 1% Triton-X, 150 mM NaCl, 1% deoxycholic acid, 2 mM PMSF. Cell lysates were equilibrated to 10% TCA with 50 µg bovine serum albumin and 0.01% sodium azide and incubated on ice for 30 min. Samples were applied to glass fiber filters, washed twice with ice cold 10% TCA and 100% ethanol, respectively. The [^35^S]-cysteine/methionine-labeled proteins were measured by scintillation or visualized by SDS PAGE. Gag or Actin proteins were collected by IP using 1 µl of Gag antibody [32] or actin antibody (Abcam ab6276) on Protein A Sepharose beads (GE Healthcare). After SDS PAGE, [^35^S]-cysteine/methionine-labeled proteins were detected by Phosphorimager analysis as described previously [33].

### RNP immunoprecipitation and immunoblot

CEMx174 cells (1×10^7^) were washed twice with PBS and harvested on ice in lysis buffer supplemented with 100 U of RNaseOUT (Invitrogen) as described previously [34]. IP were performed on Protein A Sepharose beads that had been conjugated to CBP80 (Bethyl A 301–794A, 5 µg) or eIF4E (Santa Cruz sc-9976, 5 µg) antiserum prior to incubation with cytoplasmic extract (250 µg). After overnight incubation at 4°C, complexes were washed twice with low salt RIPA buffer (50 mM Tris pH 8.0, 0.1% SDS, 1% Triton-X, 150 mM NaCl, 1% deoxycholic acid) plus 2 mM PMSF and 2 µl/ml RNaseOUT (Invitrogen); followed by one wash with high salt RIPA buffer (50 mM Tris pH 8.0, 0.1% SDS, 1% Triton-X, 1 M NaCl, 1% deoxycholic acid) plus 2 mM PMSF and 2 µl/ml RNaseOUT. IP efficiency was assessed by immunoblotting of equivalent aliquots of input lysate and washed bead slurry (15 µl). RNA was extracted from the immunoprecipitates using Trizol (Invitrogen) following manufacturer's protocol.

The antibodies used for immunoblotting were: CBP80 (Bethyl A301–793A), eIF4E (Cell Signaling 9742), GRP78 (Abcam ab21685), α-tubulin (Santa Cruz sc-23948), HA.11 (Covance MMS-101R), HIV-1 Vif (6459, NIH AIDS reagent program), PARP (Cell Signaling 9542), β-Actin (Abcam ab6276), phospho-eIF2α Ser51 (Cell Signaling 3597), total eIF2α (Cell Signaling 9722), phospho-eIF4E Ser209 (Cell Signaling 9741), total eIF4E (Cell Signaling 9742), phospho-4E-BP1 Ser65 (Cell Signaling 9451), total 4E-BP1 (Cell Signaling 9452), phospho-Mnk1 Thr197/202 (Cell Signaling 2111), total Mnk1 (Cell Signaling 2195), Caspase-3 (Cell Signaling 9662) and CBP80 (Bethyl A301–793A).

### RNA analyses

The reverse transcription (RT) reactions were carried out using the Omniscript reverse transcriptase (Qiagen), random hexamers and 100 ng of cellular RNA or equivalent volumes of RNA eluant from the RNA-IP samples. Ten-percent of the RT reaction was subjected to real-time PCR quantification on Lightcycler (Roche) or standard PCR amplification followed by electrophoresis on 1.5% agarose and detection by ethidium bromide. Thirty cycles of PCR were performed using the following primers and annealing temperatures: HIV-1 gag forward KB1614 5′-GTAAGAAAAAGGCACAGCAAGCAGC and HIV-1 gag reverse, KB1615 5′-CATTTGCCCCTGGAGGTTCTG; 57°C. nef forward KB1330 5′-CGGCGACTGGAAGAAGCG and nef reverse KB1331 5′-CTCGGGATTGGGAGGTGGGTC; 58°C. gapdh forward KB1371 5′-CATCAATGACCCCTTCATTGAC and gapdh reverse KB1372 5′-CGCCCCACTTGATTTTGGA; 52°C. The RNase protection assay (RPA) was performed as described previously [35], [36].

### Statistical analyses

Sample size of three observations per treatment provided more than 95% power to detect differences in the experiments performed in this study. Treatment groups consisted of three or more independent biological replicate samples. As previously established [34], significant differences among groups or treatments were measured by one-way ANOVA models; multiple comparisons of the treatments to the mock control used Dunnett simultaneous tests; pairwise comparisons employed Tukey's method. Log transformation was performed on the outcome variable and two-sided tests were used to calculate probability (*p*-value).

## Results

### Suppression of cellular translation is attributable to HIV-1-induced cell cycle arrest

**Figure 1:**
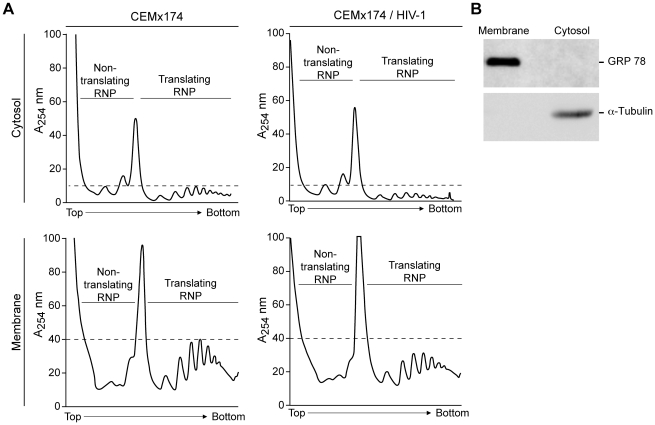
HIV-1 infection correlates with a subtle decrease in soluble and membrane-associated polyribosomes. (A) Representative ribosomal RNA profile of mock or HIV-1-infected CEMx174 cells after sucrose gradient centrifugation and detection of the rRNA by absorbance at 254 nm. Labels designate the non-translating ribonucleoprotein complexes (RNPs) and adjacent peaks are 40S, 60S and 80S ribosome, respectively. The translating RNP contains the polyribosomes. (B) Immunoblot with endoplasmic reticulum marker, GRP78 and cytosolic marker, α-Tubulin showed effective fractionation of the membrane-associated and soluble cytosolic compartments of the infected CEMx174 cells.

To examine the impact of HIV-1 infection on the translational activity of lymphocytes, a series of complementary experiments were performed. Previously, ribosomal RNA profile analysis revealed a subtle change in the polyribosome profile of HIV-infected lymphocytes, suggesting a reduction in cellular translation [37]. To revisit this observation, sucrose gradient were performed and ribosomal RNA profiles were generated on soluble ribosomes and membrane-associated ribosomes. In comparison to uninfected CEMx174 cells, HIV-1 infected cells displayed a subtle reduction in the amplitude of polyribosome peaks (Figure 1A, Cytosol, compare area under the dotted line), recapitulating the published results [37]. Furthermore, the difference was observed in both the soluble- and membrane-associated ribosomal RNA profiles (Figure 1A, compare Cytosol and Membrane). Immunoblotting with Tubulin and GRP78 verified the effective fractionation of soluble and membrane-associated compartments (Figure 1B). The results suggested that the translation initiation process was suppressed by HIV-1 infection.

**Figure 2:**
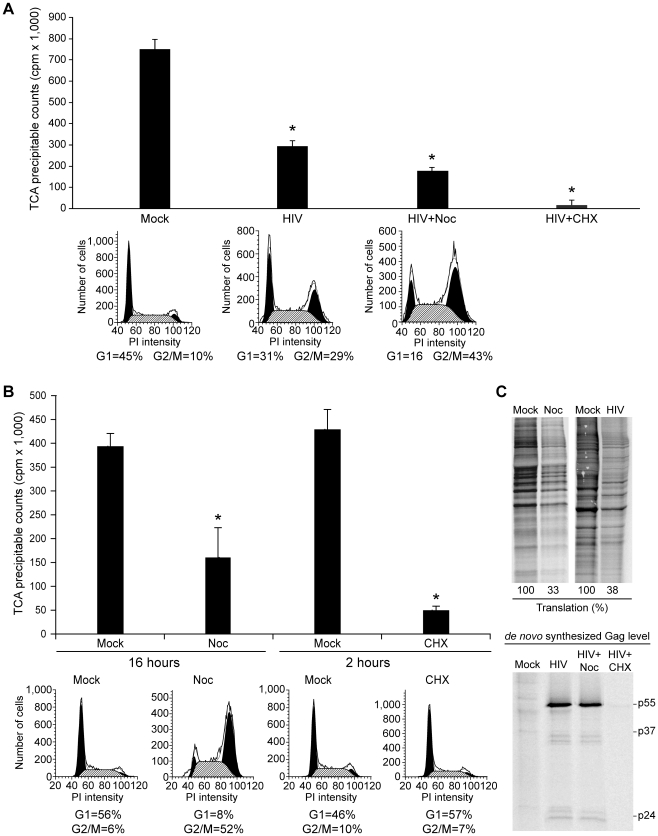
HIV-1 suppresses lymphocyte translation but HIV-1 gag translation is sustained. (A) Following HIV-1 infection and treatment with nocodazole (Noc) or cyclohexamide (CHX), CEMx174 cells were incubated with [^35^S]-cysteine/methionine for one hour and proteins were precipitated with TCA. [^35^S]-cysteine/methionine incorporation was measured by scintillation in three independent experiments and is presented with standard deviation. Asterisk indicates the significant reduction, *p<0.05*. Propidium iodide staining intensity and ModFit analysis demonstrates the number and percentage of cells in the G1 or G2/M phase of the cell cycle. Percentages are summarized below the plots. (B) CEMx174 cells received mock, nocadozole or CHX treatment. TCA precipitable counts and the cell cycle profiles were measured as described in (A). (C) Equivalent whole-cell extracts were resolved by SDS-PAGE and labeled proteins were detected by the phosphorimager analysis. Percentage cellular translation relative to the mock treatment or infection is summarized below the gel. Equivalent whole-cell extracts were subjected to immunoprecipitation after metabolic labeling and proteins were resolved by SDS-PAGE and phosphorimager analysis is presented. Positions of Gag p55, p37 and p24 are indicated.

Next metabolic labeling experiments were performed on HIV-1^NL4-3^ infected CEMx174 cells. In parallel, aliquots of infected cells were also treated with cycloheximide, which inhibits polypeptide chain elongation during translation [38], or nocadozole, which arrests cells in the G2/M phase of the cell cycle [39]. The cells were labeled with ^35^S-methionine/cysteine for one hour, proteins were precipitated, and the relative levels of *de novo* protein synthesis were measured by scintillation. In comparison to mock-infected cells, the ^35^S-methionine/cysteine incorporation was significantly lower in HIV-1-infected cells (*p*<0.0001) (Figure 2A, compare Mock to HIV-1, reduction by 60%), indicating that HIV-1 infection reduces *de novo* protein synthesis. The addition of nocadozole to uninfected cells produced a similar reduction in *de novo* protein synthesis (*p*<0.0001) (Figure 2B, compare Mock to Noc, reduction by 60%). As expected, protein synthesis was eliminated by cycloheximide (*p*<0.0001) (Figure 2B, compare Noc to CHX).

The addition of nocadozole to HIV-1-infected cells exacerbated the reduction in cellular translation (*p*<0.0003) (Figure 2A, compare HIV-1 to HIV-1+Noc, reduction by 60% and 75%, respectively). The small cumulative decrease observed may be attributed to the effect of nocadozole on the uninfected cells, or an alternative inhibitory mechanism that augments HIV-1 induced translation suppression. As expected, the addition of CHX to HIV-1-infected cells eliminated cellular translation (*p*<0.0001) (Figure 2A, compare HIV-1 to HIV-1+CHX), indicating that HIV-1 infection reduces but does not completely shutdown host protein synthesis. To correlate HIV-1-induced cell cycle arrest with the protein synthesis activity of the cells, fluorescent activated cell sorting (FACS) of PI-stained cells was performed. As expected, HIV-1 infection increased the proportion of cells in the G2/M phase relative to mock-infected cells (Figure 2A). The addition of nocadozole to HIV-1-infected cells further increased the proportion of cells in the G2/M phase (Figure 2A). By comparison, the addition of nocadozole to uninfected cells increased the proportion of cells in the G2/M phase of the cell cycle relative to untreated cells in correlation with the reduction in protein synthesis (Figure 2B).

Metabolic labeling followed by SDS-PAGE and autoradiography of nocadozole-treated or HIV-1-infected cells showed equivalent reductions in cellular translation in comparison to mock-treated or mock-infected cells, respectively (Figure 2C *top panel*). Parallel immunoprecipitation (IP) of HIV-1 Gag evaluated the levels of *de novo* Gag synthesis after nocadozole or cycloheximide treatment (Figure 2C *bottom panel*). The results demonstrate that *de novo* Gag synthesis was sustained upon nocadozole treatment and eliminated in the presence of cycloheximide.

**Figure 3:**
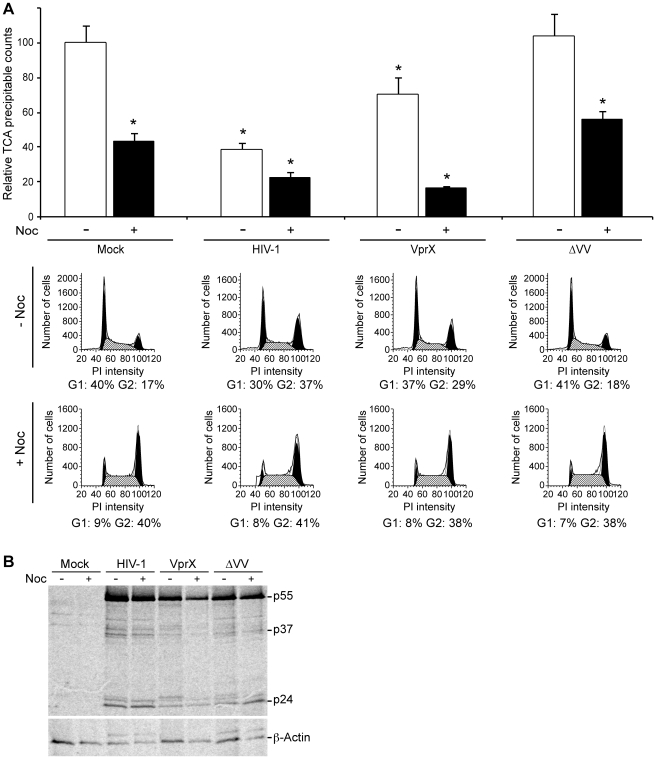
HIV-1 accessory proteins Vpr and Vif are necessary for HIV-1-induced suppression of cellular translation. (A) CEMx174 cells infected with HIV-1, VprX, ΔVifVprX (ΔVV) or mock-infected were incubated with or without nocodazole (Noc) and then metabolically labeled with [^35^S]-cysteine/methionine for one hour and proteins were precipitated with TCA. [^35^S]-cysteine/methionine incorporation was measured by scintillation in three independent experiments and is presented with standard deviation. Asterisk indicates the significant reduction (*p<0.05*) as summarized in text for all of the infections except ΔVifVprX (*p* = 0.8062). Nocadozole treatment significantly reduced the *de novo* protein synthesis in all of the infections. Propidium iodide staining intensity and ModFit analysis demonstrates the number and percentage of cells in the G1 or G2/M phase of the cell cycle. Percentages are summarized below the plots. (B) Comparison of *de novo* HIV-1 Gag and cellular Actin protein production with or without nocadozole treatment. CEMx174 cells were metabolically labeled with [^35^S]-cysteine/methionine for one hour, followed by immunoprecipitation with antisera to Gag and Actin. The immunoprecipitated proteins were resolved by SDS-PAGE and phosphorimager analysis is presented. Positions of Actin and Gag p55, p37 and p24 are indicated.

Based on the similarity in the effect of HIV-1 infection and nocadozole treatment on total protein synthesis, we investigated whether HIV-1-induced cell cycle arrest is necessary for the observed suppression of host translation. HIV-1-induced cell cycle arrest has been attributed to the HIV-1 accessory proteins Vpr and Vif and ablation of the *vpr* and *vif* open reading frames is necessary to eliminate G2/M arrest [40], [41], [42]. CEMx174 cells were infected with HIV-1 or derivative viruses deficient in *vpr* (VprX) or *vpr* and *vif* (ΔVifVprX). FACS results demonstrated that the HIV-1 infected cells were arrested in the G2/M phase, whereas a partial G2/M arrest was observed for VprX-infected cells, recapitulating published work that the deletion of *vpr* alone is not sufficient to eliminate G2/M arrest (Figure 3A). Consistent with published results, G2/M arrest was not observed in ΔVifVprX-infected cells. The metabolic labeling results determined that the cellular protein synthesis was suppressed in cells infected with HIV-1 (*p*<0.0001), but not ΔVifVprX (*p* = 0.8062) (Figure 3A, compare white bars). A partial suppression in protein synthesis was observed for cells infected with VprX (*p*<0.0001). As expected, nocadozoletreatment significantly suppressed translation in all viral infections relative to the mock-infected cells (Figure 3A, compare black bars). Metabolic labeling and IP results determined that the Gag protein synthesis was sustained independent of the virus used and was not reduced by nocadozole treatment (Figure 3B). Consistent with the reduction in polyribosomes observed in Figure 1A, *de novo* Actin synthesis was reduced during HIV-1-infection and correlated with the cell cycle arrest phenotype (Figure 3B).

Additional pulse-chase and Gag IP experiments investigated whether Gag protein stability differed amongst the virus derivatives. The results determined that the Gag protein half-life was similar in each of the viral infections (Figure S1). Consequently, HIV-1 induces a generalized suppression of host protein synthesis due to virus- induced cell cycle arrest, but Gag protein stability is unaffected.

**Figure 4:**
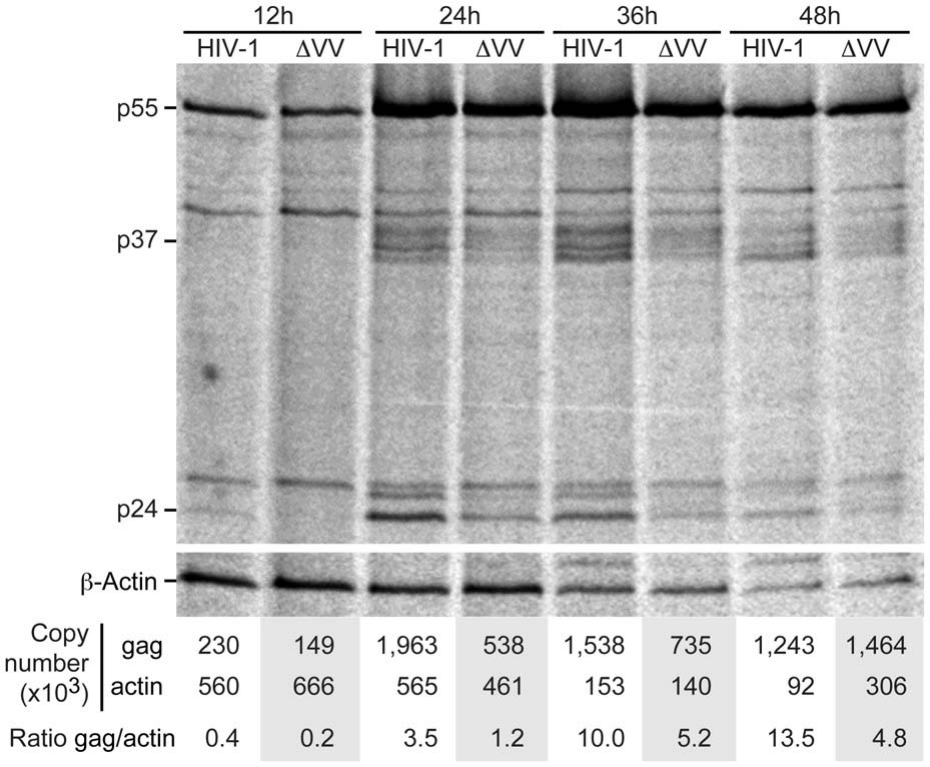
HIV-1 gag mRNA translation is resistant to suppression of global cellular translation. CEMx174 cells were infected with HIV-1 or ΔVifVprX (ΔVV) and evaluated at 12 hour intervals post-infection. At indicated intervals, replicate cultures were metabolically labeled with [^35^S]-cysteine/methionine for one hour and proteins immunoprecipitated with antisera to Gag and Actin. The immunoprecipitated proteins were resolved by SDS-PAGE and phosphorimager analysis is presented. Positions of Actin and Gag p55, p37 and p24 are indicated. In parallel, RNA was harvested and RT-qPCR with gag or actin primers was performed to measure RNA copy numbers. The ratio of the RNA copy numbers is presented.

To evaluate changes in Gag translation efficiency during G2/M arrest, we analyzed gag mRNA translation during the course of HIV-1 infection. Four independent replicate infections with HIV-1 or ΔVifVprX were performed and representative results are presented. HIV-1 or ΔVifVprX infections generated readily detectable Gag protein by 12 h, and Gag protein synthesis increased as the infections progressed (Figure 4). Aliquots of cells were harvested for RNA isolation and RT-qPCR was performed with *gag* and *actin* primers. During HIV-1 infection, the levels of gag RNA peaked at 24 hour (Figure 4). Likewise actin protein and mRNA levels peaked at 24 hour and declined in parallel with gag mRNA levels. We attribute this trend to the declining cell viability over the course of the HIV-1 infection. In the ΔVifVprX infection, the gag mRNA levels continued to increase over the course of the infection (Figure 4). The relative level of Gag protein synthesized to gag mRNA was similar between HIV-1 and ΔVifVprX. The results indicated that the expression of Vpr and Vif does not significantly attenuate nor bolster the efficiency of Gag protein synthesis. In sum, the HIV-1 protein synthesis is sustained during virus-induced suppression of host translation.

### Vpr-dependent cell cycle arrest is necessary and sufficient to suppress cellular translation

**Figure 5:**
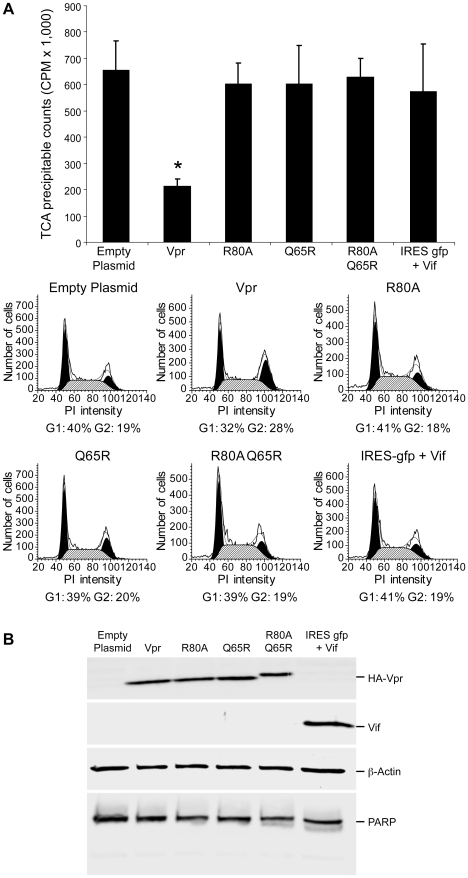
Vpr is sufficient for the cell cycle arrest and suppression of cellular translation. (A) HEK 293 cells were transfected with IRESgfp (Empty plasmid), bicistronic VprIRESgfp expression plasmid that encodes Vpr or indicated substitution mutants, or IRESgfp plus Vif expression plasmid. GFP-positive cells were sorted and metabolically labeled with [^35^S]-cysteine/methionine for one hour, followed by TCA precipitation and scintillation. The histogram presents average TCA precipitable counts of the incorporated [^35^S] and the standard deviation from three independent experiments. Asterisk indicates a significant reduction in *de novo* protein synthesis (*p = 0.0019*) in response to Vpr. Propidium iodide staining intensity and ModFit analysis determined the number and percentage of cells in the G1 or G2/M phase of the cell cycle. Percentages are summarized below the plots. (B) Equivalent whole-cell extracts were immunoblotted with HA antibody to detect epitope tagged Vpr, and antiserum against Vif, PARP and β-Actin, respectively.

Exogenous expression of Vpr is sufficient to induce cell cycle arrest and this effect is attributed to polyubiquitination and degradation of a yet-to-be-identified substrate protein [24], [43], [44]. To determine whether or not Vpr is sufficient for suppression of cellular translation, HEK293 cells were transfected with an epitope-tagged Vpr expression plasmid. Ectopic expression of HA-Vpr was sufficient to suppress cellular protein synthesis (Figure 5A, factor of 3, p<0.002,). Flow cytometry determined that the cells were arrested at the G2/M phase (Figure 5A). HA immunoblot verified the expression of HA-Vpr (Figure 5B).

Vpr-dependent cell cycle arrest is attributable to its interaction with the Cul4A^DDB1/DCAF1^ E3 ligase and a yet-to-be-identified substrate for proteosome-mediated degradation [43], [45], [46], [47], [48], [49], [50]. Genetic analyses have shown that the Q65R mutation eliminates interaction with the DCAF1 subunit of the cullin 4a/DDB1 E3 ubiquitin ligase while the R80A mutation sustains DCAF1 interaction, but exhibits a loss of activity due to a defective interaction with a yet-to-be defined host protein. Cells transfected with the Vpr mutants R80A, Q65R or R80AQ65R [43] lacked the G2/M arrest and did not suppress protein synthesis (Figure 5A). Likewise, ectopic expression of Vif was not sufficient for G2/M arrest and did not reduce protein synthesis (Figure 5A). Immunoblotting verified equivalent expression of each protein (Figure 5B). Taken together, the results indicated that the ectopic expression of Vpr is sufficient to suppress cellular translation by inducing cell cycle arrest.

### HIV-1 infection reduces active eIF4E and increases the inhibitory form of 4E-BP1

**Figure 6:**
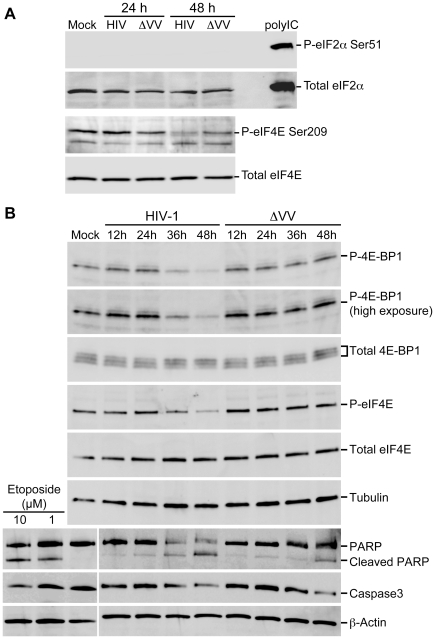
Suppression of cellular translation is attributable to HIV-1-dependent dephosphorylation of eIF4E and 4E-BP1. (A) HEK 293 cells were transfected with HIV-1^NL4-3^ or ΔVifVprX (ΔVV) for 24 or 48 hour, as indicated. Equivalent whole-cell extracts were immunoblotted with antiserum against phospho-eIF2α Ser51, total eIF2α, phospho-eIF4E Ser209 or total eIF4E, respectively. (B) CEMx174 cells were infected with HIV-1 or ΔVifVprX (ΔVV) and evaluated at 12 hour intervals. Equivalent whole-cell extracts were immunoblotted with the indicated antiserum.

To address the underlying mechanism of HIV-1 translation suppression, we assessed the phosphorylation status of host translation factors by immunoblot [1], [3], [13], [16], [51], [52], [53], [54], [55]. As shown in Figure 6A, treatment of HEK293 cells with double-stranded RNA (polyIC) upregulated total eIF2α and Ser51-phosphorylated eIF2α (P-eIF2α) (compare mock to polyIC). Transfection of HEK293 cells with HIV-1 or ΔVifVprX provirus did not perturb the levels of eIF2α or induce phosphorylation of eIF2α (Figure 6A). However, expression of HIV-1 but not ΔVifVprX reduced the abundance of Ser209-phosphorylated eIF4E (P-eIF4E), independent of the changes in the total eIF4E levels (Figure 6A, compare 24 hour and 48 hour). Evaluation over 12 hour intervals determined that the abundance of P-eIF4E and Ser65-phosphorylated 4E-BP1 (P-4E-BP1) were reduced by HIV-1 infection (see 36 hour), but not ΔVifVprX infection (Figure 6B, compare HIV-1 to ΔVV). Furthermore, HIV-1 and ΔVifVprX infections did not perturb the total protein levels of eIF4E and 4E-BP1. Uniform sample loading was confirmed by α-Tubulin immunoblot. The results demonstrated that the post-translationally modified forms of eIF4E and 4E-BP1 were reduced in the presence of HIV-1. PARP cleavage and reduction in the full-length caspase-3 were observed by 48 hour, similar to the etoposide treatment (Figure 6B, *lower panels*, compare etoposide to HIV-1 and ΔVV) [56]. The onset of PARP cleavage and caspase-3 activation did not correspond to a change in the phosphorylated forms or total amounts of eIF4E or 4E-BP1. In summary, the results suggest that the molecular basis of HIV-1 translation suppression is the downregulation of eIF4E function.

**Figure 7:**
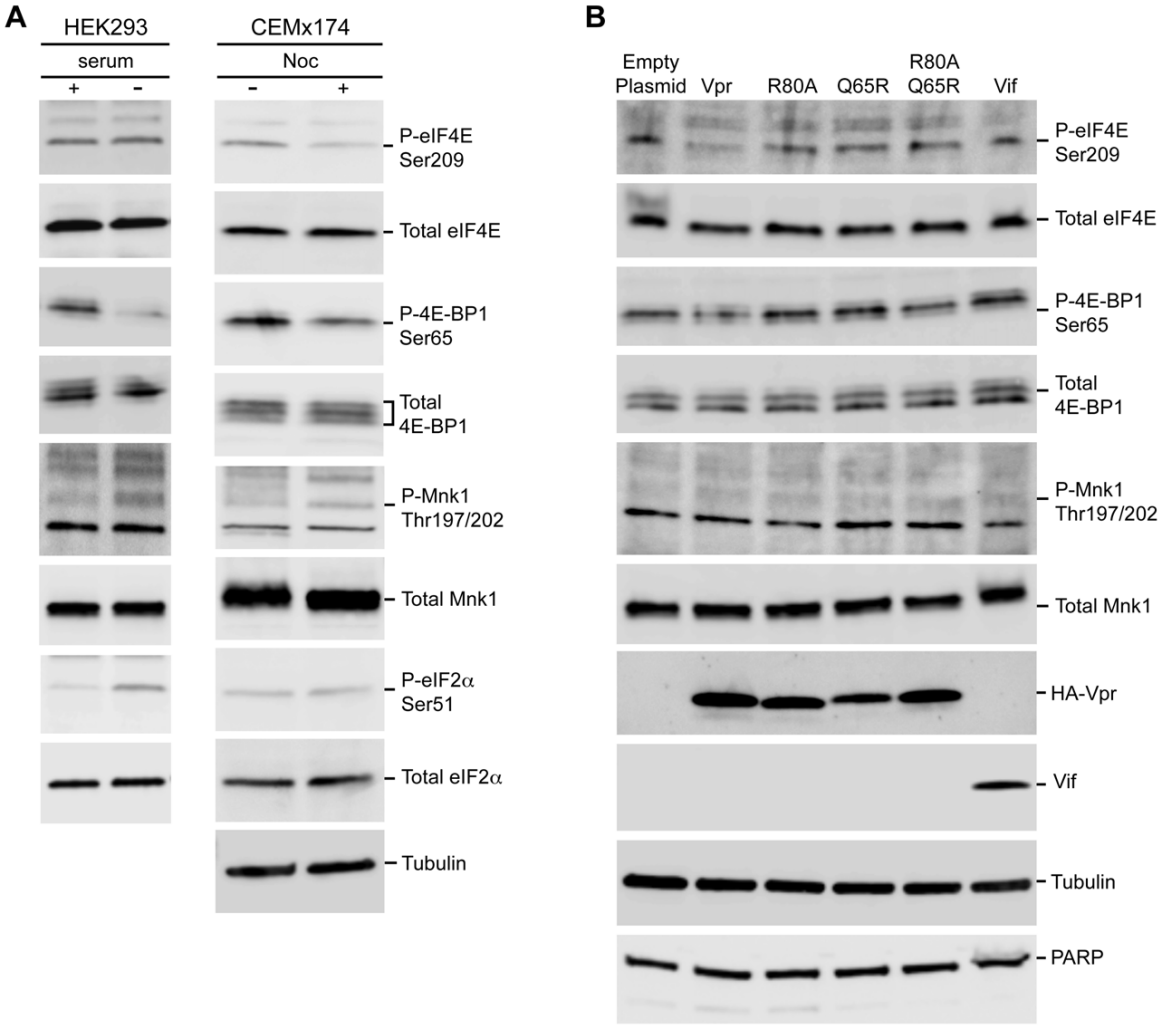
Vpr expression is sufficient to reduce the levels of phosphorylated eIF4E and 4E-BP1. (A) Serum deprivation or pharmacological cell cycle arrest is sufficient to reduce accumulation of phosphorylated eIF4E and 4E-BP1. HEK 293 cells were incubated in complete medium (serum +) or in low serum medium (−serum) for 24 hour. Equivalent whole-cell extracts were harvested and immunoblotted with phospho-eIF4E Ser209, total eIF4E, phospho-4E-BP1 Ser65, total 4E-BP1, phospho-Mnk1 Thr197/202, total Mnk1, phospho-eIF2α Ser51 and total eIF2α, antibodies respectively *(left panel)*. CEMx174 cells were incubated in complete medium that lacked or contained nocodazole (Noc) for 24 hour, and equivalent whole-cell extracts were immunoblotted with the indicated antiserum. (B) HEK 293 cells were transfected with expression plasmids encoding Vpr, indicated Vpr substitution mutant, or Vif, and whole-cell extracts were immunoblotted with the indicated antiserum.

To determine whether or not Vpr-induced cell cycle arrest is sufficient for the impairment of eIF4E activity, HEK293 cells were transfected with HA-Vpr or the Vpr mutants deficient in cell cycle arrest. The samples were evaluated relative to serum deprivation, which downregulates translation by reducing the phosphorylated form of selected translation factors [57], [58], [59]. Consistent with previous reports, serum deprivation reduced and increased the levels of P-4E-BP1 and P-eIF2α, respectively, without affecting the total protein levels (Figure 7A, compare + serum to − serum). By comparison, nocadozole treatment of CEMx174 cells reduced P-4E-BP1 and P-eIF4E, but not P-eIF2α (Figure 7A, compare + Noc to − Noc). Both serum deprivation and nocadozole treatment increased the levels of Thr197/202-phosphorylated Mnk1 (P-Mnk1) (Figure 7A, compare − serum to + Noc).

Ectopic expression of Vpr was sufficient to reduce the levels of P-4E-BP1 and P-eIF4E (Figure 7B, compare Vpr to Empty plasmid). The reduction was not observed when Vif or the Vpr mutants deficient in G2/M arrest were expressed (Figure 7B, compare Vpr to R80A, Q65R, R80AQ65R and Vif). Moreover, Mnk1 levels were unaffected and P-Mnk1 was not detected (Figure 7B), indicating that the underlying mechanism for Vpr-mediated translation suppression is distinct from serum deprivation (nutritional stress) or nocadozole treatment. Future investigation of the possible roles of Mnk2 and other components of the mTOR pathway is warranted. Equivalent expression of HA-tagged Vpr proteins and Vif was verified by HA and Vif antisera, respectively. The exogenous expression of Vpr did not affect PARP levels indicating that the downregulation of the active eIF4E was not attributable to apoptosis or poor cell viability (Figure 7B) [60], [61]. These results indicate that the downregulation of translation by Vpr is associated with reduced accumulation of P-eIF4E and P-4E- BP1.

### HIV-1 gag RNA remains bound to nuclear cap binding protein in the cytoplasm

**Figure 8:**
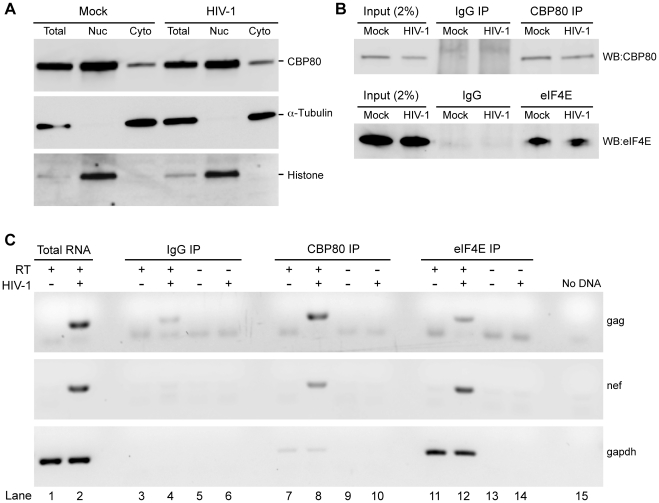
Cytoplasmic HIV-1 gag mRNA retains interaction with CBP80 in the cytoplasm. (A) CEMx174 cells infected with HIV-1 or mock-infected were used to harvest total cellular protein (Total) and the nuclear (Nuc) and cytoplasmic (Cyto) compartments. Equivalent aliquots were immunoblotted with the indicated antiserum. (B) Cytoplasmic lysates from (A) were subjected to immunoprecipitation with CBP80 *(top)* or eIF4E *(bottom)* antibodies. Immunoblotting of equivalent aliquots of input and the immunoprecipitated material determined that the IP efficiency was similar between the lysates. Specificity of the IP was verified by using IgG isotype control. (C) Immunoprecipitates from (B) were used to harvest RNA, which were subjected to reverse transcription reactions with or without reverse transcriptase (RT + or −), followed by gene-specific PCR. Products were separated by agarose gel electrophoresis. The gag, nef or GAPDH amplification products from control Total RNA preparation (lanes 1, 2) and RNA-immunoprecipitates (lanes 3–15) are indicated. Lane 15 (No DNA) indicates PCR reaction contained water in place of cDNA.

Our observation of sustained Gag protein synthesis despite reduced eIF4E activity suggested that the gag mRNP circumvents downregulation of eIF4E-dependent translation and may interact with the translational apparatus in a distinct fashion. One hypothesis is that the gag RNP uses the nuclear CBC to initiate translation. The CBC components, CBP80 and CBP20, are normally subject to competitive binding by importin-beta, which drives their nuclear reimport and efficient exchange for eIF4E [19], [62]. To assess whether HIV-1 infection alters CBC nucleo-cytoplasmic distribution, sub-cellular fractionation and immunoblots with antisera to CBP80, α-tubulin and histone H1 were performed. The results demonstrated that the abundance of CBP80 was similar between mock and HIV-1 infected cells (Figure 8A, compare Mock to HIV-1). As expected, CBP80 and histone H1 were prominent in the nucleus and α-Tubulin was prominent in the cytoplasm. Furthermore, HIV-1 infection did not affect cytoplasmic abundance of CBP80 (Figure 8A, compare Mock to HIV-1). To investigate the interaction of CBC and eIF4E with HIV-1 gag RNA, mRNPs were immunoprecipitated from cytoplasmic fractions. Immunoblots confirmed CBP80 and eIF4E were specifically immunoprecipitated with their respective antisera but not with the isotype-control IgG (Figure 8B, compare CBP80 and eIF4E to IgG). RNA was harvested from cells or the co-precipitates and subjected to reverse transcription and PCR with primers complementary to *gag*, *nef*, or *GAPDH*. As expected, gag and nef RNAs were observed solely in the RNA preparations from HIV-1 infected cells (Figure 8C, lanes 1, 3, 7, 11), whereas GAPDH RNA was observed in both mock- and HIV-1-infected cells (Figure 8C, lanes 1–2). Consistent with expectations, GAPDH RNA exhibited prominent association with eIF4E (Figure 8C, compare lanes 7 and 8 to 11 and 12). In contrast, the HIV-1 transcripts exhibited a differential pattern of eIF4E association. Unspliced gag RNA was abundant in the CBP80 RNP, while multiply spliced nef RNA was abundant in the eIF4E RNP (Figure 8C, compare lanes 8 and 12). The isotype control IgG IP lacked detectable levels of nef and GAPDH RNA (Figure 8C, lanes 3–6) or showed background levels of gag RNA (Figure 8C, lane 4). No amplification products were detected in the absence of RT (RT−) (Figure 8C, lanes 5, 6, 9, 10, 13, 14) or cDNA (Figure 8C, lane 15, water control). The results demonstrate that the cytoplasmic HIV-1 gag mRNA is predominantly associated with CBC, whereas nef and GAPDH mRNAs are predominantly associated with eIF4E. In sum, the spliced HIV-1 mRNA undergoes CBC exchange for eIF4E, while unspliced HIV-1 mRNA remains associated with the CBC in the cytoplasm.

### CBP80 is a component of the translating HIV-1 gag RNP

**Figure 9:**
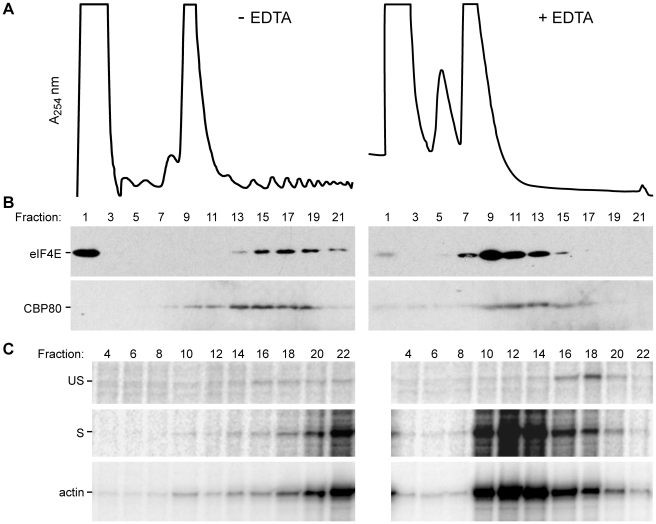
CBP80 and eIF4E co-sediment with the polyribosomes. (A) HEK293 cells were transfected with HIV-1^HxBruR−/RI−^ for 48 hour, cytoplasmic extracts were harvested, incubated in buffer with or without EDTA, and separated on 15–47.5% linear sucrose density gradients. Representative A_254_ ribosomal RNA profiles are indicated. (B) Lysates from every other fraction were immunoblotted with CBP80 and eIF4E antibodies. (C) RNA was isolated and subjected to RNase protection assays with ^32^P-labeled antisense RNA probe complementary to the HIV-1 5′ UTR and gag or actin, respectively. Detection products are the unspliced HIV-1 RNA (US), spliced HIV-1 transcripts (S) and cellular actin RNA.

A possible mechanism by which unspliced HIV-1 mRNA circumvents downregulation of eIF4E-dependent translation is the use of the CBC-associated mRNAs to engage the translation apparatus. To test this hypothesis, HEK293 cells were transfected with HIV-1^HxBruR−/RI−^ provirus and cytoplasmic extracts were prepared and fractionated on 15–45% sucrose gradients [30]. Replicate sucrose gradients were performed in the presence of EDTA, which dissociated the polysomes and produced an upward shift of ribosomal RNAs in the gradients (Figure 9A, fractions 13–21, compare −EDTA to +EDTA). Alternating gradient fractions were used to extract protein (odd number) and RNA (even number). Immunoblots demonstrated that eIF4E is in both the nontranslating mRNPs (Figure 9B, fraction 1, −EDTA) and polysome fractions (Figure 9B, fractions 13–21, −EDTA). EDTA treatment induced an upward shift of eIF4E and eIF4E was most abundant in the 80S ribosomal fraction (Figure 9B, fraction 9, +EDTA). In comparison, CBP80 was not seen in nontranslating mRNPs (Figure 9B, fraction 1, −EDTA), but was detected in the 80S ribosomal fractions (Figure 9B, fractions 9–11, −EDTA) and the fractions containing polysomes (Figure 9B, fractions 13–17, −EDTA). The EDTA treatment produced an upward shift in the CBP80 that was less prominent than the shift in eIF4E (Figure 9B, compare −EDTA to +EDTA). The shifted CBP80 became detectable in the nontranslating RNPs (Figure 9B, fractions 1–5, +EDTA) and was most abundant in the 80S ribosomal fractions (Figure 9B, fractions 9–11, +EDTA). Taken together, these results demonstrate that CBP80 bound RNPs are predominately engaged in translation.

RPAs were used to examine the distribution of HIV-1 and actin mRNAs across the same sucrose gradients. The HIV-1 RNA probe used is complementary to the 5′ splice site of HIV-1 and hence measured the abundance of unspliced gag transcript (US) and spliced viral mRNAs (S) (inclusive of the singly-spliced 4 kb and multiply-spliced 2 kb HIV-1 mRNAs) [36], [37]. The actin RPA probe served to control for the minor variations in the sample processing. As shown in Figure 9C, all probed mRNAs were predominately found in the polysome fraction of the gradient. Treatment with EDTA produced an upward shift in the HIV-1 RNAs, demonstrating that the sedimentation of the RNAs was dependent on the interaction with ribosomes (Figure 9C, fractions 16–22, −EDTA). In summary, the results indicate that the CBC-bound HIV-1 gag mRNA undergoes active translation.

### CBP80 is a component of HIV-1 virions

**Figure 10:**
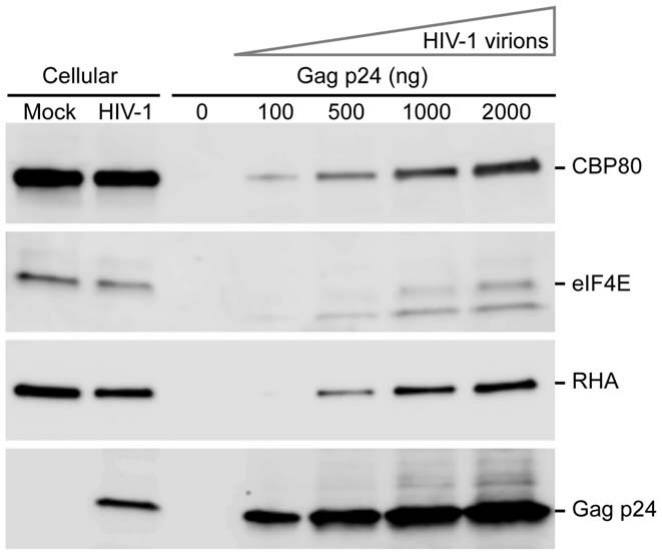
CBP80 is incorporated into HIV-1 virions. Cellular protein and cell-free supernatant medium from CEMx174 cells or CEMx174/HIV-1 were harvested and subjected to immunoblot. Virions in the medium were collected by ultracentrifugation, subjected to Gag ELISA and the indicated aliquots (0 to 2000 ng) were evaluated with the indicated antisera.

The observation that the HIV-1 unspliced mRNPs retains association with the CBC during translation raised the possibility that CBC interaction is retained during encapsidation. To investigate this possibility, virions were harvested and subjected to Gag ELISA and equivalent virion RNPs were subjected to immunoblot. Immunoblots of cell-associated proteins from uninfected or HIV-1-infected CEMx174 cells showed no change in abundance or size of CBP80 and eIF4E (Figure 10, compare Mock to HIV-1). Parallel testing of purified virions detected both CBP80 and eIF4E (Figure 10, right panel showing HIV-1 virions). Parallel testing of cell-free medium harvested from the uninfected cells eliminated the possibility of contamination with cellular material (Figure 10, compare 0 ng to 2000 ng Gag). Notably, an additional polypeptide was detected by eIF4E antiserum, which is smaller and ∼2-fold more abundant than eIF4E. The identity of this polypeptide will be determined in the future. Equivalent loading of virion preparation was verified using antiserum to known virion components, host RNA helicase A (DHX9) [33] and capsid protein, Gag p24. These results demonstrate that the CBC remains associated with the HIV-1 unspliced RNA undergoing encapsidation.

## Discussion

Our study of HIV-1-host interaction has generated new and fundamental insight into the translational control of HIV-1 mRNAs and the HIV-1 infected cell. Using multiple approaches, we demonstrated that the host cell translation is suppressed during acute HIV-1 infection, an effect associated with reduced levels of phosphorylated eIF4E and 4E-BP1. The changes in protein modification and translation are in response to the cell cycle arrest induced by the viral accessory protein Vpr. Exogenous expression of Vpr is sufficient for global translation suppression and mutations that ablate Vpr-induced cell cycle arrest eliminate this effect. Despite the reduction in total protein synthesis, HIV-1 sustains translation of its structural and enzymatic proteins. We hypothesize that this effect is attributable to the unique composition of the viral RNP, in particular the retention of its interaction with the nuclear cap binding complex (CBC) throughout its lifetime in the cytoplasm. These findings outline a novel viral strategy to circumvent downregulation of eIF4E-dependent translation, a mechanism that might also be exploited by selected cellular mRNAs during conditions of cellular stress. The retention of CBC on the mRNA allows for continued viral mRNA translation when global translation is reduced by limited eIF4E function, such as during cellular stress response, induction of apoptosis, and progression from the G2/M phase of the cell cycle [63]. We hypothesize that CBC-bound HIV-1 mRNAs and selected host mRNAs represent a novel class of RNA regulon [64] whose protein products need to be synthesized during conditions of limited eIF4E activity.

Previous studies have determined that CBC is required for the nuclear export of U snRNA to the cytoplasm [65], [66] and U snRNP assembly [67]. The U snRNA is tethered to the CRM1 nuclear export receptor by the phosphorylated adapter for RNA export (PHAX) protein [68]. In the case of incompletely spliced HIV-1 RNAs, the tethering function is provided by the virus-encoded adapter protein Rev. Rev interacts with the cis-acting Rev responsive element (RRE) in the env open reading frame and mediates the export of the unspliced viral RNAs to the cytoplasm, in a CRM1-RanGTP-dependent manner [21], [69]. In contrast, completely spliced viral mRNAs utilize NXF1/NXT1, the same adaptor proteins used by the spliced cellular mRNAs. Once in the cytoplasm, the RNP undergoes rearrangement, part of which involves the exchange of the CBC for cytoplasmic eIF4E. The CBC is then reimported into the nucleus by importin-α [67].

Biophysically, CBC and eIF4E employ two aromatic side chains to interact with the 7-methylguanine of the bound mRNA in an induced-fit structure [70]. However, the CBC has significantly greater affinity for the 7-methylguanosine cap structure than eIF4E [71]. Interaction with the translation initiation cofactor eIF4G is necessary to provide eIF4E with a competitive advantage for binding 7-methylguanosine cap structure [72]. However, recent work by Yedavalli and Jeang determined that cytoplasmic unspliced HIV-1 RNA has a tri-methlyguanosine cap, making these RNAs structurally distinct from cellular mRNAs, which have a mono-methylguanosine cap [73]. The difference in RNA cap structure is a function of the export pathway used since the same RNA sequence transported via NXF1/NXT1 lacked the tri-methlyguanosine modification. We hypothesize that the distinct modifications of the 5′-cap generates a unique molecular signature that dictates the fate of this mRNA template during G2/M phase, cellular stress that limits eIF4E activity, and/or stimulation of the innate immune response [74]. Biophysical measurements have determined that the CBC has a ∼100 fold higher affinity for tri-methylguanosine relative to mono-methylguanosine [71]. Such differences in affinity may provide a mechanism for preferential retention of the CBC by HIV-1 gag mRNA allowing it to circumvent reduced eIF4E-dependent translation, sustain viral protein synthesis and remain bound during virion assembly. Selective translation of HIV-1 structural protein mRNAs may also be facilitated by a distinct set of interactions that mediate translation initiation of the CBC-bound mRNAs. A recent study determined that CBC-bound mRNAs had a selective advantage in engaging the translational apparatus [75] perhaps as a result of using different factors (CTIF versus eIF4G) to support translation [76]. The possibility that CBC retention is a result of the presence of an IRES within HIV-1 unspliced RNA was also considered. To address this issue, we evaluated HIV-1 Rev-dependent env RNA that lacks the previously mapped IRES [77], [78], [79]. As shown in the supplementary material (Figures S2 and S3), the env transcript that lacks the IRES maintained the preferential retention of CBC and the spliced product was associated with eIF4E.

The differences by which HIV-1 mRNAs engage the translational machinery may be important to allow HIV-1 to subvert NMD [80]. Both unspliced and singly spliced HIV-1 RNAs have multiple termination codons throughout the sequence that would normally signal degradation by NMD. Other retroviruses, such as the Rous sarcoma virus, use an RNA stability element to antagonize recognition of premature termination codons [81], [82]. In a similar fashion, the distinct composition of the HIV-1 RNPs may serve to antagonize the induction of the NMD, by preventing recognition of termination codons present in the incompletely spliced and unspliced HIV-1 mRNAs.

Taken together, the data presented in this report indicate that the unspliced and singly spliced HIV-1 transcripts have a distinct pattern of regulation from the bulk of host mRNA in the cytoplasm, conferred, in part, by differences in the composition of the RNPs. Such differences permit HIV-1 to maximize viral protein synthesis and virion production under conditions that reduce cell survival. Our discovery also opens new avenues to investigate the mechanistic control of CBC/eIF4E cap exchange that is fundamental to translational regulation in all metazoan cells.

## Supporting Information

Figure S1 **Gag proteins with similar stability are expressed from HIV-1 and the derivatives viruses.** CEMx174 cells were infected with HIV-1, ΔVifVprX (ΔVV) or VprX for 48 hour. The infected cells were metabolically labeled with [^35^S]-cysteine/methionine for one hour, washed and incubated for indicated intervals after the addition of complete medium. Equivalent volumes of cells were subjected to immunoprecipitation and proteins were resolved by SDS-PAGE and phosphorimager analysis is presented. Positions of Gag p55, p37 and p24 are indicated.
(TIF)

Figure S2 **CBP80 and eIF4E co-sediment with polysomes in cells transfected with subgenomic HIV-1 env plasmid.** (A) HEK 293 cells were transfected with pgTat and pCMVRev for 48 hour, cytoplasmic extracts were harvested and incubated with or without EDTA, and applied to 15–47.5% linear sucrose density gradients. Representative A_254_ ribosomal RNA profiles are indicated. (B) Lysates from every other fraction were immunoblotted with CBP80 and eIF4E antibodies. (C) RNase protection assays of RNA isolated from every other fraction. Designations indicate the unspliced (US) reporter transcript; spliced (S) env RNA; and cellular actin RNA.
(TIF)

Figure S3 **Unspliced pgTat mRNA coprecipitates with CBP80 and the spliced env transcript coprecipitates with eIF4E.** (A) HEK 293 cells were transfected with pgTat and pCMVRev for 48 hour. Equivalent cytoplasmic lysates were immunoprecipitated with the isotype control (IgG), eIF4E or CBP80 antibodies. (B) Graphical representation of pgTat indicating the position of the RPA probe used (top). RNase protection assays of RNA isolated from the immunoprecipitations. Designations are unspliced (US) pgTat transcript and spliced (S) env transcript.
(TIF)
