# Supplementary figures and images for: Thriving under Stress: Selective Translation of HIV-1 Structural Protein mRNA during Vpr-Mediated Impairment of eIF4E Translation Activity
Source: PLoS Pathog. 2012 Mar 22;8(3):e1002612. doi: 10.1371/journal.ppat.1002612 (PMC3310836; doi:10.1371/journal.ppat.1002612)

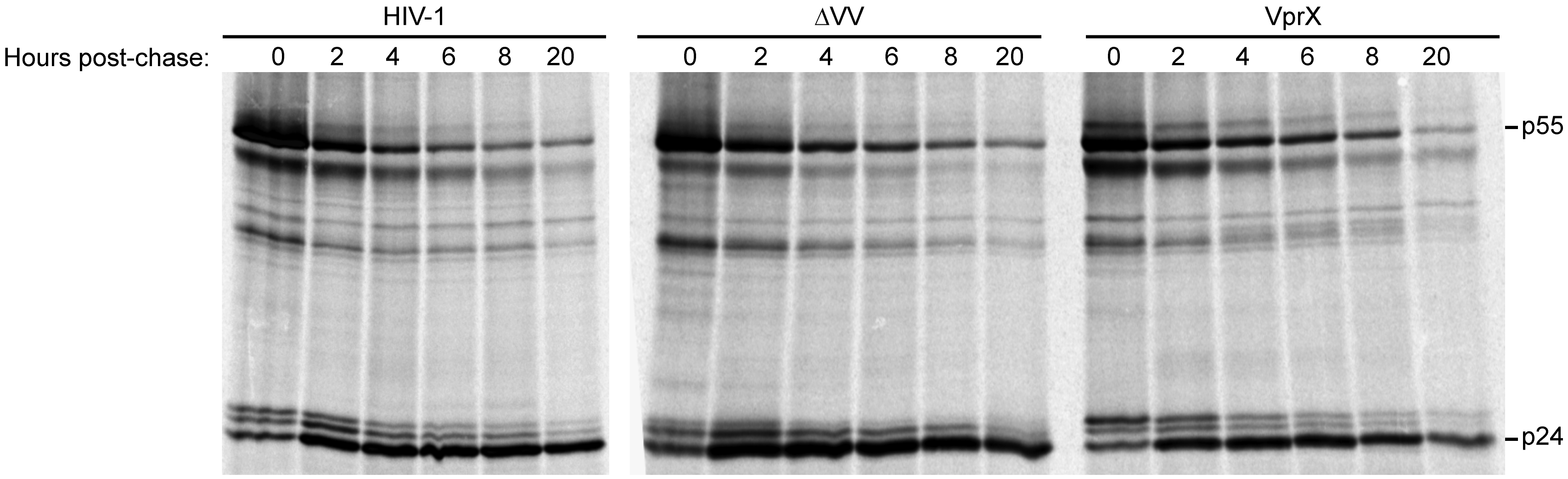

Supplement: Figure S1 — Gag proteins with similar stability are expressed from HIV-1 and the derivatives viruses. CEMx174 cells were infected with HIV-1, ΔVifVprX (ΔVV) or VprX for 48 hour. The infected cells were metabolically labeled with [35S]-cysteine/methionine for one hour, washed and incubated for indicated intervals after the addition of complete medium. Equivalent volumes of cells were subjected to immunoprecipitation and proteins were resolved by SDS-PAGE and phosphorimager analysis is presented. Positions of Gag p55, p37 and p24 are indicated. (TIF) [file ppat.1002612.s001.tif]

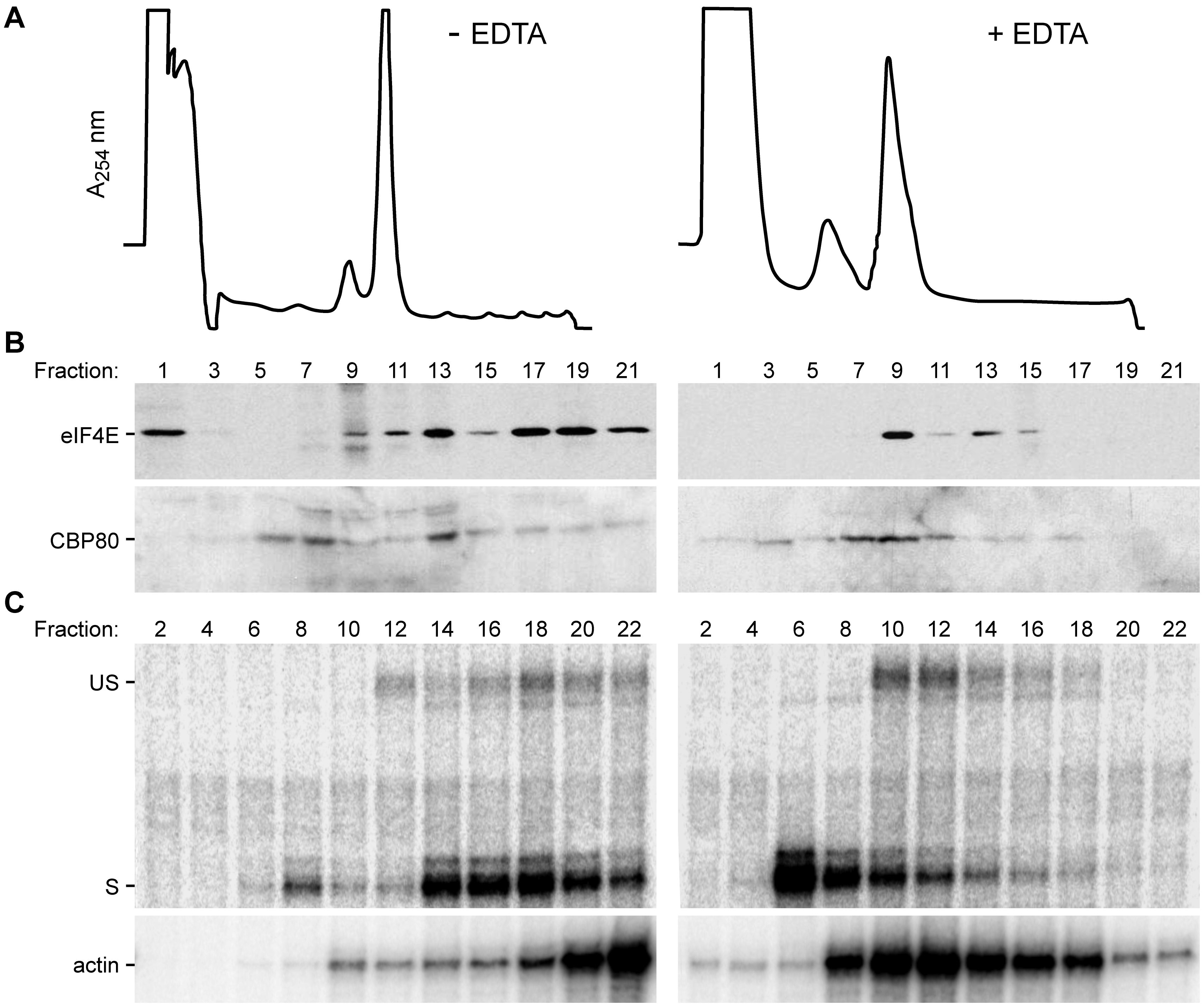

Supplement: Figure S2 — CBP80 and eIF4E co-sediment with polysomes in cells transfected with subgenomic HIV-1 env plasmid. (A) HEK 293 cells were transfected with pgTat and pCMVRev for 48 hour, cytoplasmic extracts were harvested and incubated with or without EDTA, and applied to 15–47.5% linear sucrose density gradients. Representative A254 ribosomal RNA profiles are indicated. (B) Lysates from every other fraction were immunoblotted with CBP80 and eIF4E antibodies. (C) RNase protection assays of RNA isolated from every other fraction. Designations indicate the unspliced (US) reporter transcript; spliced (S) env RNA; and cellular actin RNA. (TIF) [file ppat.1002612.s002.tif]

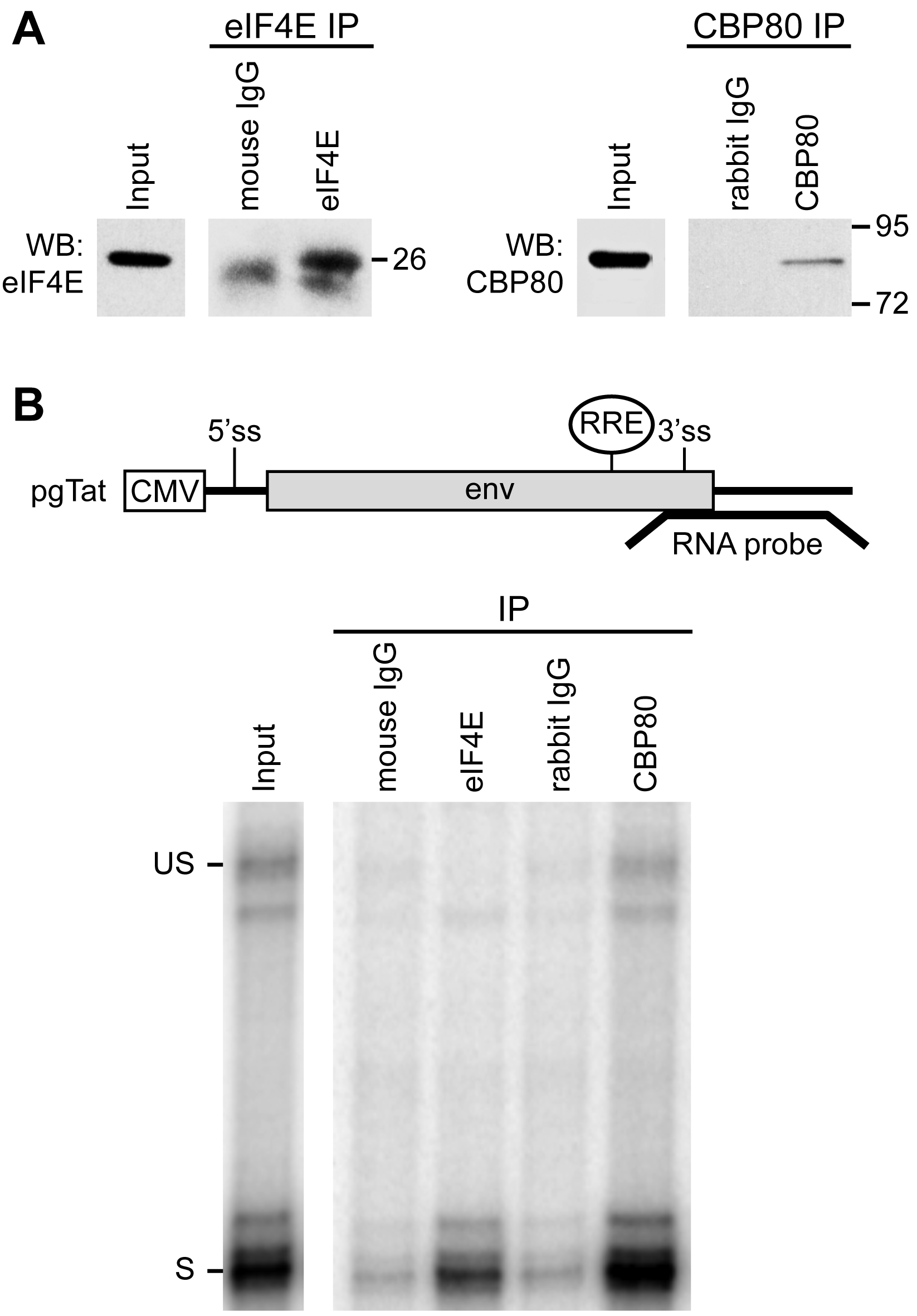

Supplement: Figure S3 — Unspliced pgTat mRNA coprecipitates with CBP80 and the spliced env transcript coprecipitates with eIF4E. (A) HEK 293 cells were transfected with pgTat and pCMVRev for 48 hour. Equivalent cytoplasmic lysates were immunoprecipitated with the isotype control (IgG), eIF4E or CBP80 antibodies. (B) Graphical representation of pgTat indicating the position of the RPA probe used (top). RNase protection assays of RNA isolated from the immunoprecipitations. Designations are unspliced (US) pgTat transcript and spliced (S) env transcript. (TIF) [file ppat.1002612.s003.tif]
